# Pathological Findings, Clinical Characteristics, and Outcomes of Thyroid Lymphomas: A Single Tertiary Care Hospital Experience

**DOI:** 10.7759/cureus.82610

**Published:** 2025-04-19

**Authors:** Reema M Alqadiri, Bushra Aljuhani, Saeed Alshieban, Majed Pharaon

**Affiliations:** 1 Anatomic Pathology, King Abdulaziz Medical City, Riyadh, SAU; 2 Pathology and Laboratory Medicine, King Abdulaziz Medical City, Riyadh, SAU

**Keywords:** burkitt lymphoma, diffuse large b-cell lymphoma (dlbcl), mucosa-associated lymphoid tissue (malt) lymphoma, plasmacytoma lymphoma, primary thyroid lymphoma (ptl)

## Abstract

Background: Primary thyroid lymphoma (PTL) is a rare malignancy accounting for a small percentage of thyroid cancers and extra-nodal lymphomas. Treatment and prognosis depend on the histologic type and stage. Local studies and international reviews highlight the predominance of B-cell non-Hodgkin's lymphomas. This study aims to identify the subtypes, clinical characteristics, and outcomes of PTL in a tertiary care hospital.

Methodology: This study presents a case series of 12 patients diagnosed with PTL between 2010 and 2024 at King Abdulaziz Medical City (KAMC) in Riyadh, Saudi Arabia. Descriptive statistics were used to summarize cohort characteristics. Continuous variables were presented as mean and standard deviation. Categorical variables were represented as frequencies and percentages.

Results: Among the 12 patients included with PTL, histopathological examination identified 66.7% with diffuse large B-cell lymphoma (DLBCL), followed by mucosa-associated lymphoid tissue (MALT) lymphoma in 16.7% of cases. Half of the cases had thyroiditis, and the majority presented with neck swelling and compressive symptoms. Most had lymph node involvement (66.67%).

Conclusion: In summary, this study provides an exclusive insight into the pathological findings, clinical characteristics, and outcomes of PTL in a tertiary care hospital. PTL is a rare malignancy; however, it is a potential diagnosis when encountering an enlarged neck mass, especially with Hashimoto’s thyroiditis. Overall, DLBCL was the most common type identified. The majority of the cases had cervical/mediastinal lymph node involvement and thyroiditis.

## Introduction

Primary thyroid lymphoma (PTL), while uncommon, presents a diagnostic and therapeutic challenge for clinicians due to its overlap with other thyroid conditions [1]. Despite its rarity, standing for only 0.5-5% of thyroid malignancies and less than 3% of extra-nodal lymphomas, PTL poses unique complexities arising from its origin within the thyroid gland [2,3].

Several studies have shed light on various aspects of PTL, offering valuable insights into its clinical presentation, management, and outcomes. For instance, a 2012 case series highlighted the prevalence of neck swelling and diffuse large B-cell lymphoma (DLBCL) among seven diagnosed patients, while also suggesting a better prognosis for mucosa-associated lymphoid tissue (MALT) lymphoma [4]. This aligns with broader literature that shows B-cell lymphomas, particularly DLBCL and MALT, as the most common subtypes of PTL [5-7].

Accurate diagnosis is often challenging due to PTL's mimicry of other thyroid pathologies [1,8,9]. Some studies have proposed diagnostic algorithms involving ultrasound, aspiration cytology, and flow cytometry to improve accuracy [7,10]. However, the importance of careful histopathological examination and immunohistochemical analysis has been emphasized for the definitive diagnosis [9,11].

Treatment strategies for PTL vary depending on factors such as the patient's age, lymphoma subtype, and stage [12]. Recent studies highlight the need for individualized treatment plans for managing PTL in specific patient populations [13,14]. Chemotherapy, often combined with radiotherapy or immunotherapy, remains the mainstay of treatment [5].

Despite growing knowledge, further research is necessary to optimize PTL diagnosis, treatment, and ultimately patient outcomes. This case series of 12 patients diagnosed with PTL at a tertiary care hospital aims to contribute to a more comprehensive understanding of the disease in a specific patient population. By analyzing the pathological findings, clinical characteristics, and treatment outcomes of these cases, we hope to identify the most common types of PTL found in our region and contribute valuable data for local healthcare providers.

## Materials and methods

This case series study was conducted at the Department of Pathology, King Abdulaziz Medical City (KAMC), Ministry of National Guard Health Affairs, Riyadh, Saudi Arabia. It encompassed a detailed review of pathological slides within the Department of Pathology and an examination of patient charts through the electronic health information system (BestCare, ezCareTech, Seoul, South Korea). Ethical approval was obtained from the ethical committee of King Abdullah International Medical Research Center (KAIMRC) with Institution Review Board (IRB) number IRB/2686/23, which takes into consideration patients’ privacy and confidentiality. Informed written consent has been obtained for participation and publishing patient details while ensuring patient anonymity is meticulously protected throughout the manuscript.

This case series study included all patients, regardless of age or gender, diagnosed with PTL between the years 2010 and 2024. Patients exhibiting secondary thyroid involvement by lymphoma were excluded from the study to maintain the focus on PTL cases.

Data were collected through the examination of histological preparations, including hematoxylin and eosin (H&E) and immunohistochemical (IHC) stain slides, retrieved from our archives. A panel of IHC stains (IHC) was done on formalin-fixed, paraffin-embedded (FFPE) samples using the avidin-biotin-peroxidase complex, Ventana BenchMark Ultra (Roche Diagnostics, Basel, Switzerland). The panel used is different between individual cases based on the possible differential diagnosis, and the overall IHC used includes CD45, CD20, PAX-5, CD3, CD5, CD10, BCL6, BCL2, MUM1, CD30, CD38, CD138, kappa, lambda, Ki-67, and c-Myc, in addition to in situ hybridization for Epstein-Barr virus (EBV) and EBV-encoded small RNA (EBER). All cases were re-examined microscopically by a certified hematopathologist to confirm diagnoses and classification of lymphoma. A structured data collection sheet was used by co-investigators to gather relevant clinical and pathological data from each patient's medical record using the BestCare system.

Upon completion of data collection, all information was revised for accuracy and completeness before statistical analysis. A descriptive statistical analysis was conducted on the dataset to summarize the demographic characteristics of the participants, including age, gender, and other features, providing an overview of the 12 patients. Clinical and pathological factors such as presenting symptoms, type of imaging, biopsy, lymphoma subtype, treatment, and outcome were also statistically analyzed. All statistical analyses were conducted using SPSS version 29.0.0 software (IBM Corp., Armonk, NY).

## Results

Demographics of patients with primary thyroid lymphoma

In this study, 12 patients with PTL were included. All patients were Saudi nationals, with ages ranging from 30 to 92 years (median: 67.50 years; mean: 67.83 years). Out of the 12 patients, seven were female (58.33%), and five were male (41.67%) (Table 1).

**Table 1:** Characteristics, histopathological subtypes, and outcomes of 12 cases of PTL. PTL: primary thyroid lymphoma; NGC: non-germinal center; GC: germinal center; SOB: shortness of breath; NOS: not otherwise specified; R-CHOP: rituximab, cyclophosphamide, doxorubicin, vincristine, and prednisolone.

	Age, gender	Presenting clinical symptoms	Lymph node involvement site	Type of biopsy	Histopathological type of PTL	Autoimmune disease	Type of treatment	Outcome	Relapse number of times, site	Duration of follow-up in years	Remission
Case 1	77, Female	Neck mass	No lymph node involved	Total thyroidectomy	Diffuse large B-cell lymphoma, NGC	Lymphocytic Hashimoto thyroiditis	Chemotherapy (6 cycles), total thyroidectomy	Alive	No	0.3	Yes
Case 2	64, Male	Neck swelling, dysphagia, voice change	Enlarged superior mediastinal lymph nodes	Partial resection (debulking thyroidectomy)	Plasmacytoma/multiple myeloma	Thrombocytopenia, Hashimoto thyroiditis	Chemotherapy (6 cycles), radiotherapy, partial thyroidectomy	Alive	Yes, 1 time, larynx	2.9	Yes
Case 3	30, Female	Neck mass	Right level IIA lymph node	Total thyroidectomy	Burkitt lymphoma	No autoimmune disease	Chemotherapy, total thyroidectomy	Alive	No	1.2	No
Case 4	67, Male	Neck mass, stridor	Cervical and mediastinal lymph nodes	Total thyroidectomy	Diffuse large B-cell lymphoma, NGC	No autoimmune disease	Chemotherapy (6 cycles of R-CHOP), thyroidectomy	Alive	No	5.0	Yes
Case 5	77, Female	Neck mass	No lymph node involved	Total thyroidectomy	Diffuse large B-cell lymphoma, GC	Lymphocytic Hashimoto thyroiditis	Chemotherapy (6 cycles R-CHOP), debulking thyroidectomy	Dead	Yes (1 time), new bilateral neck pathological lymph nodes	5.0	Yes
Case 6	66, Female	SOB, neck swelling, cough	No lymph node involved	Needle core biopsy	Diffuse large B-cell lymphoma, GC	Lymphocytic Hashimoto thyroiditis	Chemotherapy (6 cycles of R-CHOP), left hemithyroidectomy	Alive	No	10	Yes
Case 7	59, Male	Goiter	Left cervical lymph node enlargement	Total thyroidectomy	Diffuse large B-cell lymphoma and follicular lymphoma, grade 3A	Thyroiditis	Chemotherapy (6 cycles of R-CHOP), total thyroidectomy, hypothyroidism on levothyroxine 125	Alive	No	0.1	Yes
Case 8	72, Female	SOB, noisy breathing, large thyroid mass	Size prominent left cervical lymph nodes, unchanged left level 2A lymph node	Total thyroidectomy	Extranodal marginal zone lymphoma of mucosa-associated lymphoid tissue	Lymphocytic Hashimoto thyroiditis	Total thyroidectomy	Alive	No	0.8	Yes
Case 9	80, Female	Neck swelling, SOB, hoarseness of voice, dysphagia, weight loss	Cervical and mediastinal lymph nodes	Needle core biopsy	Diffuse large B-cell lymphoma, GC	No autoimmune disease	Chemotherapy(mini R-CHOP 3 cycles), open tracheostomy (tumor debulking)	Alive	No	2.5	No
Case 10	62, Female	Multinodular goiter	No lymph node involved	Total thyroidectomy	Diffuse large B-cell lymphoma, NOS, germinal center subtype (80%) and follicular lymphoma, grade 3B (20%)	No autoimmune disease	Chemotherapy (R-CHOP 6 cycles), total thyroidectomy	Alive	No	3.4	Yes
Case 11	92, Male	Neck mass, dysphagia, SOB, voice change	Multiple prominent mediastinal lymph nodes	Needle core biopsy	Diffuse large B-cell lymphoma, GC	No autoimmune disease	Did not receive treatment because not fit for any chemotherapy	Dead	No	0.2	No
Case 12	68, Male	SOB, increased work of breathing, Progressive stridor	Right level IIa/III/IVa lymph nodes	Needle core biopsy	Extra-nodal marginal zone lymphoma of mucosa-associated lymphoid tissue	No autoimmune disease	Chemotherapy (2 cycles of R-CHOP)	Alive	No	0.1	No

Clinical characteristics

As presented in Table 1, most patients presented with neck swelling/mass (91.67%), and over half with compressive symptoms (58.33%). In addition, half of the cases had a history of Hashimoto's thyroiditis autoimmune disorder.

As for lymph node involvement, eight cases (66.67%) had secondary lymph node involvement. The most common sites were cervical lymph nodes, followed by mediastinal lymph nodes.

Moreover, seven cases had a total thyroidectomy, four had a core needle biopsy, and one case had a partial resection.

Histopathological subtypes

**Figure 1:**
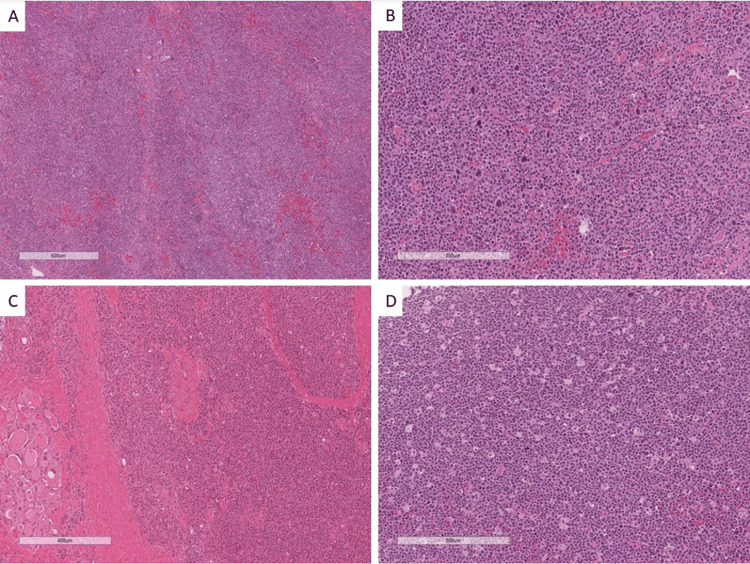
Different types of hematolymphoid tumors involving the thyroid gland. (A) Mucosa-associated lymphoid tissue (MALT) lymphoma, (B) diffuse large B-cell lymphoma (DLBCL), (C) plasmacytoma, and (D) Burkitt lymphoma (hematoxylin and eosin, original magnification 100x).

**Figure 2:**
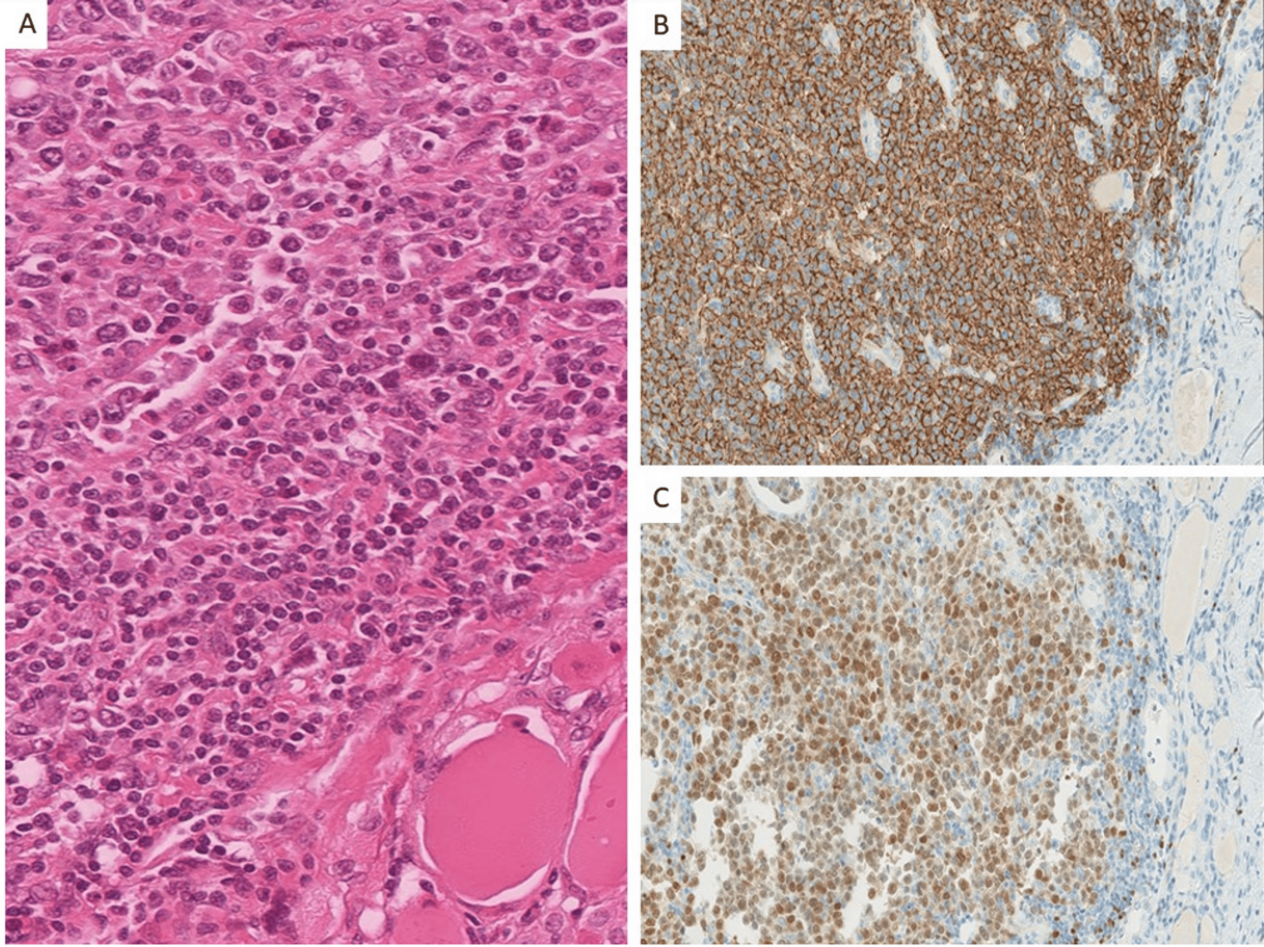
A case of diffuse large B-cell lymphoma (DLBCL) showing large atypical lymphoid cells with few entrapped thyroid follicles in the lower right corner. (A) Hematoxylin and eosin (original magnification, 400x). The atypical lymphoid cells are immunoreactive to (B) CD20 and (C) PAX-5, confirming a B-cell phenotype (original magnification, 200x).

**Figure 3:**
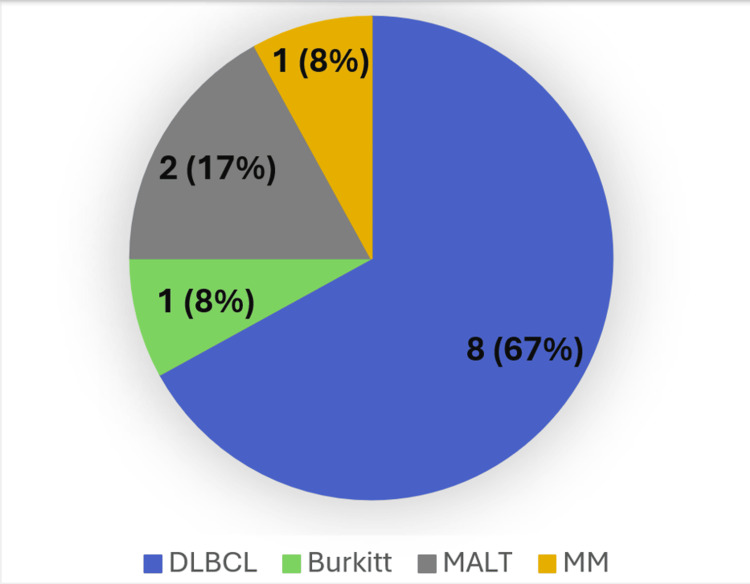
Distribution of primary thyroid lymphoma in 12 patients. DLBCL: diffuse large B cell lymphoma; MALT: mucosa-associated lymphoid tissue; MM: multiple myeloma.

Under the microscope, histopathological examination showed DLBCL as the most common subtype (66.7%), followed by MALT lymphoma (16.7%). Notably, one patient each presented with Burkitt lymphoma and plasmacytoma/multiple myeloma, as shown in Figures 1-3.

Radiology

As for imaging modalities, neck CT and PET-CT scans were performed in 83.3% of the patients. In addition, 16.7% of the patients had a neck MRI performed, as seen in Table 2.

**Table 2:** Radiology imaging modalities performed.

Neck CT			PET-CT			Neck MRI	
	Frequency	%	Frequency	%	Frequency		%
No	2	16.67	2	16.67	10		83.33
Yes	10	83.33	10	83.33	2		16.67

Treatments and outcomes

Eleven patients (91.7%) received treatment, with chemotherapy being the mainstay (83.3%), and radiotherapy and levothyroxine in some cases. The average number of chemotherapy cycles was 5.22 (Table 1).

In terms of outcomes, 10 patients were alive at the last follow-up, while two patients died from the disease. Two patients (20% of those with available data) experienced relapse, and eight patients (88.9% of those with available data) achieved complete remission, with an average remission duration of 3.14 years. The duration of follow-up ranged from 0.1 to 10 years, with a median follow-up of 2.5 years (Table 1).

## Discussion

Thyroid lymphoma is a rare type of thyroid cancer and can be classified into primary or secondary thyroid lymphoma [15]. PTL usually occurs with a history of pre-existing Hashimoto’s thyroiditis, and it is often diagnosed because of airway obstruction symptoms caused by the enlarging thyroid gland (goiter) [3]. In this current study, we aimed to investigate the pathological findings, clinical characteristics, and outcomes of PTL at King Abdulaziz Medical City (KAMC), Riyadh, Saudi Arabia.

This study included a total of 12 patients, with a mean age of 67.8 years, and a little over half were females (58.33%). The most common histopathological subtype was DLBCL (66.7%), followed by MALT lymphoma (16.7%). These patients usually presented with a neck swelling/mass and compressive symptoms, and the majority of cases had lymph node involvement as well.

A study done in Saudi Arabia, from 2005 to 2012, included cases of PTL and concluded that the most frequent clinical presentation was neck swelling and compression symptoms [4]. Four (57.2%) patients were of DLBCL, two (28.6%) patients had marginal zone B-cell MALT lymphomas, and one (14.2%) patient had T-cell lymphoma [4]. These patients were treated with chemotherapy and involved field radiotherapy 36-40 Gy in 18-20 fractions [4].

Another review study done in Greece on PTL showed that DLBCL is the most common subtype, followed by MALT lymphoma or a mixed type (similar to our study). Other subtypes are less common. Lymphomas derived from T-cells and Hodgkin's lymphomas are extremely rare. Hashimoto's autoimmune thyroiditis has been implicated as a risk factor for lymphoma [5,16]. At the molecular level, the Wnt5a protein and its receptor Ror2 are involved in the course of the disease. Ultrasonography, fine needle aspiration (FNA) biopsy, and core or open biopsies combined with new diagnostic facilities contribute to an accurate diagnosis [5].

One of the main limitations met in this case series is the retrospective design and the existing bias due to the small sample size.

We recommend future studies to assess PTLs' clinical characteristics, pathological subtypes, treatment, outcomes, and complications in different hospitals within different regions in the country. In addition, studies with longer follow-ups with the patients are needed.

## Conclusions

In summary, this study offers valuable insight into the pathological findings, clinical characteristics, and outcomes of PTL in KAMC, Riyadh, Saudi Arabia. PTL, while a rare malignancy, should be considered in the differential diagnosis of an enlarged neck mass. Early detection is crucial for timely intervention and improved patient management. Furthermore, given the association between Hashimoto's thyroiditis and PTL, establishing protocol-based management and follow-up for patients with Hashimoto's thyroiditis is warranted to potentially improve patient outcomes.

Overall, DLBCL is the most common type in our center, followed by MALT lymphoma. The majority of the cases had cervical/mediastinal lymph node involvement and Hashimoto's thyroiditis. Our study showed similar results to other published international data in the most common subtypes, i.e., DLBCL and MALT lymphoma.
